# Changes in species composition of South Georgia seabirds and mammals 1985–2023: decrease in opportunities for local enhancement?

**DOI:** 10.1098/rstb.2025.0363

**Published:** 2026-10-15

**Authors:** Samantha A. Monier, Lisa L. Manne, Richard R. Veit

**Affiliations:** ^1^ Biology, CUNY Graduate Center, New York, NY, USA; ^2^ Biology, CUNY College of Staten Island, Staten Island, NY, USA

**Keywords:** Antarctic birds, facilitation, foraging success, local enhancement, network connectivity, recurrent group analysis, South Georgia

## Abstract

Seabirds cue into other birds and marine mammals to amplify their foraging success. Consequently, changes to the community (e.g. as the climate warms) can impact foraging success and potentially fitness. For example, flying birds that use groups of seals or penguins as cues might decline if the seal or penguin population declines, owing to fewer opportunities for positive foraging associations with one another. To test this idea, we leveraged data from transect surveys spanning 38 years (data collected in 1985, 1991, 1993 and 2023) in waters surrounding the subantarctic island South Georgia. We asked: ‘Has the frequency with which seabirds form statistically significant pairings with other species declined over time?’ We found such changes have occurred: network connectivity (the percentage of species pairings out of all possible pairs) decreased from approximately 40 to 7%, although the overall impact of warming on the community in 2023 was complicated by the arrival of a 100 km iceberg from the Weddell Sea. Possible fitness impacts from reduced associations could have broad-ranging consequences as the ocean continues to warm and the community changes. The results presented here represent one of just a handful of at-sea surveys documenting the winter seabird community at South Georgia.

This article is part of the theme issue ‘Multi-predator feeding aggregations in pelagic waters’.

## 1. Introduction

Seabirds use conspecifics, other bird species, mammals and predatory fish as cues to the presence of prey [1–8], thus enhancing their foraging success and presumably fitness. These behaviours, known as local enhancement (LE), are common to all the world's oceans and families of seabirds and essential in tropical oceans [2,4,9]. Seabirds follow whales, dolphins and fishes, and are attracted to aggregations of predators feeding on fish or zooplankton. Indeed, black-browed albatrosses (*Thalassarche melanophris*) are more spatially correlated with other black-browed albatrosses than they are with their primary prey, Antarctic krill [7], supporting the importance of LE in that the birds use each other in preference to their prey to find prey. Further, seals and whales have recently been shown to follow seabirds [10], suggesting a mutualistic benefit for those using LE. While it is technically difficult and perhaps impossible to demonstrate that seabirds that join these feeding flocks experience higher fitness than those that don't, such feeding aggregations have been described in detail since the 1880s [11–13], and it seems most unlikely that individuals that join these aggregations do not gain some fitness benefit from doing so. Furthermore, seabird LE has been positively linked to fitness proxies, such as decreased search time [5] and increased patch detection [14], while multiple individuals foraging at the same patch has been shown to enhance prey capture [15–17] owing to prey becoming more dispersed in the water column (see [2] for a review).

At the same time, seabirds as a group have recently experienced substantial declines in population size, from the combined effects of predation from non-native mammals at breeding colonies [18,19], mortality in fishing gear, changing climate leading to reduced prey abundance [20–22], impact of industrial fishing on prey abundance [23,24], increased abundance of krill-eating whales and seals recovering from overharvesting (e.g. [25]) and a combination of other factors [23,26]. If seabirds find food by noting the behaviours of other foragers, and populations of these other foragers decline for some of the reasons just noted, then accelerated declines are likely to follow as foragers lose access to the information provided by other foragers. We are not suggesting that reduced opportunities for LE are the sole factor affecting foraging success of Antarctic seabirds. But we do argue that decreased LE can further reduce foraging success already reduced by competition with other foragers and commercial fishers. As foraging efficiency is critical for survival and reproduction, the implication is that these populations may decline at a faster rate than otherwise predicted by environmental change alone.

Such a framework is described in [27], drawing attention to an under-explored consequence of environmental change—the disruption of social networks and thus, how seabirds obtain and transfer crucial foraging information. Ecological change alters which species are present in a community, and because marine predators often forage in mixed-species groups, this affects who forages with whom and their foraging success. Using data collected off the eastern end of South Georgia from 1985–1993, Monier *et al.* [27] demonstrated that seabird associations in the region can change over time and may be related to changes in sea surface temperature. Here, we test this framework with the addition of new data collected in 2023 (returning to the same study site 30 years after what is reported in [27]).

### (a) Changes at South Georgia affecting the marine predator community

South Georgia has undergone substantial ecological change since the 1990s and will undoubtedly continue to do so [28]. While ocean temperatures have risen globally, the largest increase in heat storage has occurred in Antarctic and subantarctic waters [29]. South Georgia has warmed significantly, at a rate of approximately 0.13°C per decade from 1905–2016 (total annual mean warming air temperature at Grytviken, South Georgia = 1.42°C [30]); the subsequent glacier loss and sea level rise [31,32] will have consequences for the marine community. Rats have historically plagued seabirds on the island, and though recently eradicated, warmer temperatures will make future invasions difficult if not impossible to contain [33]. Importantly, the marine predators at South Georgia rely heavily on a single prey species, Antarctic krill (*Euphausia superba*), tying them to the abundance of this species [34–36]. Krill, however, are decreasing and shifting their ranges as a result of climate and other anthropogenic changes [37–40]. Additionally, krill stocks are impacted by the recent recovery of previously hunted marine mammal populations, with consequences for krill-dependent birds [36,41,42].

Given such ecological change in the region, we expect to find changes to the seabird and marine mammal community (implicit in which are abundance changes [43]) and hypothesize a concurrent decline in the number of interspecific foraging associations over time. To our knowledge, South Georgia's winter seabird and marine mammal community has only been described a handful of other times ([44,45] as well as individual survey reports by the British Antarctic Survey [46–51].

## 2. Methods

Our analyses are based on shipboard surveys of birds and mammals made off the eastern end of South Georgia in May to July, 1985, 1991, 1993 and 2023. On each cruise, we counted birds and marine mammals along transect surveys. To facilitate comparison between new data collected in 2023 and those of our previous study collected in 1985–1993 [27], we have replicated many of the core methods previously described in [27]—including choice of study site, data collection and treatment protocols. We collected data during all weather conditions; however, visibility was never below 300 m and generally far greater. Ship-following birds were recorded only once on each cruise, when first noted. In addition to these data from our east-end transects, we also note observations from outside the zone and from other parts of the island when stated. R.R. Veit was present on all four cruises to train all observers and ensure consistency across surveys.

### (a) Transect surveys

In addition to the surveys conducted off the east end of South Georgia in June to July of 1985 (2 days, 222.36 km surveyed), 1991 (5 days, 654.97 km surveyed) and 1993 (6 days, 720.51 km surveyed), we completed an additional 7 days of surveys between 11 and 17 July 2023 from the bridge of the R/V Laurence M. Gould (mean survey distance per side of the ship = 709.31 km—although every effort was made to survey the port and starboard sides of the ship simultaneously, occasional differences in start times between the port and starboard teams resulted in 710.75 km surveyed off the starboard side and 707.88 km surveyed off the port) (figure 1). Travelling at approximately 10 km per hour, each transect took approximately 6 hours to complete (just short of all daylight hours).

**Figure 1.**
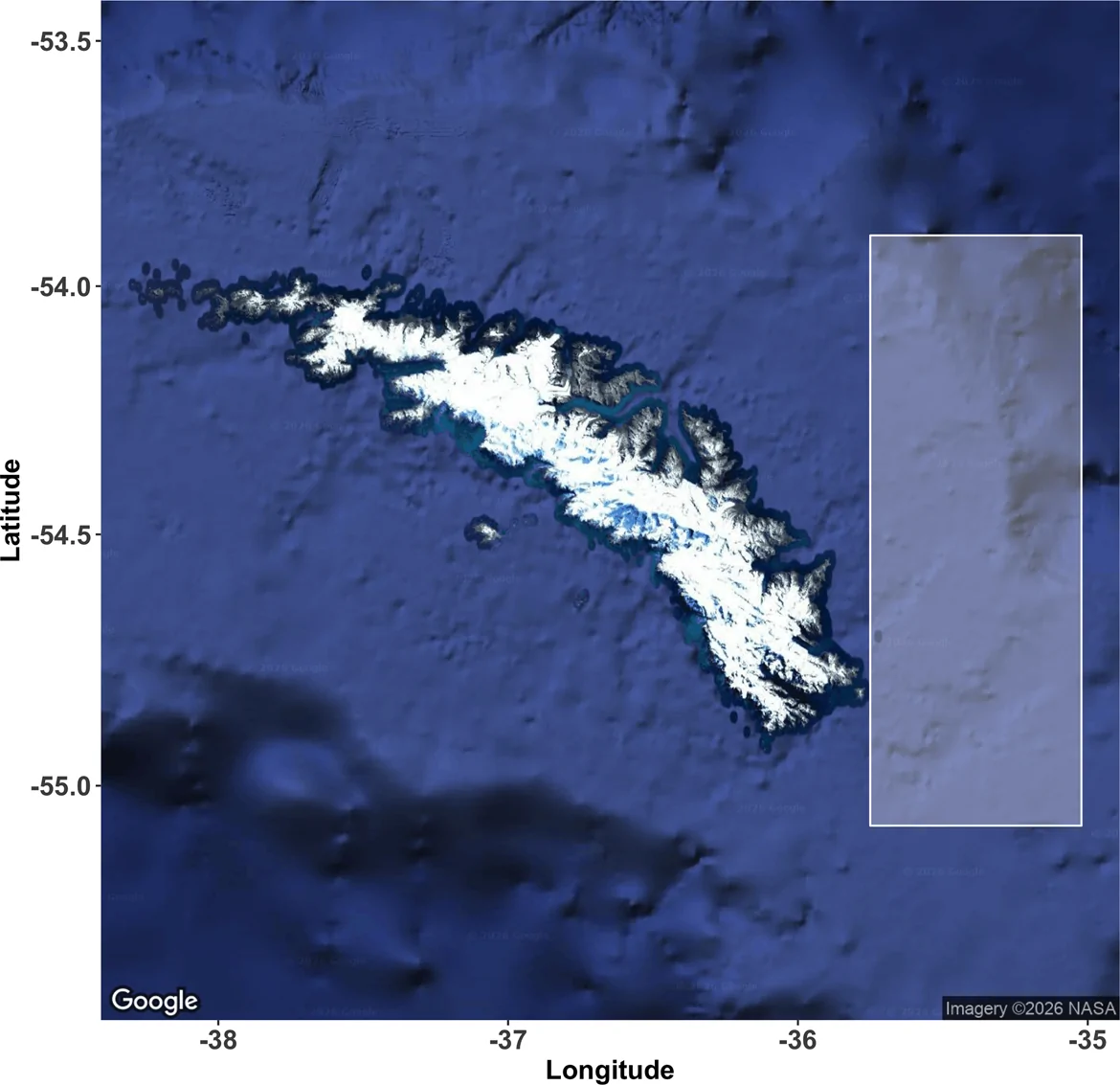
South Georgia, located just northeast of the West Antarctic Peninsula. The shaded box off the east end encompasses all transect areas surveyed by ship in 1895, 1991, 1993 and 2023. All transects were longitudinal. The map was produced in R using the package ggmap [52], with Google Map ‘satellite’ selected as the background.

 In 2023, we used distance sampling methods [53], with two teams of two (one observer and one data recorder). Each team surveyed one side of the ship. All bird and marine mammal sightings were recorded (species, count and coordinates), along with their positions and radial distance in relation to the ship. Data from the two sides of the ship were largely similar, but were averaged to eliminate possible biases between sides (see supplementary material, figure S1 for a comparison of the port and starboard observations). In 1985–1993, however, 300 m strip transects were used, and only those sightings that fell within a 300-m-wide band of the ship were recorded. This was standard procedure for shipboard seabird transects at the time [54] (i.e. before the use of distance sampling [53]). Because the 2023 survey had a wider observation field for the purpose of comparing these data with those from past surveys, the observation field was truncated *post hoc* and only sightings that fell within a 300-m-wide band of the ship were used in downstream analyses. We achieved this by converting the radial distances associated with each observation in 2023 into perpendicular distances, as in [55]. An examination of the observations excluded by this truncation revealed the vast majority of sightings fell within the 300 m strip (approx. 97% of the individuals seen off the port side as well as approx. 97% of those off the starboard). The only species to be seen exclusively outside of the 300 m strip was a single fin whale (*Balaenoptera physalus*).

Marine predators were identified to the lowest possible taxon in the field; however, data on some difficult-to-identify species were combined *post hoc*. These include: diving-petrels (mostly common diving-petrels (*Pelecanoides urinatrix*), but we could not rule out potentially some South Georgia diving-petrels (P*elecanoides georgicus*)), giant petrels (a mixture of Northern (*Macronectes halli*) and Southern giant petrels (*Macronectes giganteus*), which were not always possible to tell apart in the field), whales (a mixture of fin whales, humpback whales (*Megaptera novaeangliae*), southern right whales (*Eubalaena australis*) and a small number of whales that we were unable to identify in the field), prions (mostly Antarctic prions (*Pachyptila desolata*) but also potentially thin-billed/slender-billed prions (*Pachyptila belcheri*)) and a single unidentified dolphin.

### (b) Recurrent group analysis

We used recurrent group analysis (RGA) [56] to identify significant associations among pairs of species. RGA determines whether the number of joint occurrences between species is greater than would be expected, given the independent distributions of each species in the pair (with ‘occurrence’ defined as presence or absence in a grid cell). We reason that significant (*p* < 0.05) pairings among species reflected the use of LE; in other words, species using each other as cues would occur together significantly more often than expected owing to chance. RGA takes into account the spatial distributions of both species involved in foraging aggregations, and thereby factors out spatial association owing to similar habitat use. RGA also has the advantage of ignoring negative correlation of abundance owing to largely non-overlapping range or habitat.

For the 1985 survey, we only had the species totals per day, so we could not test for statistical association among species but still used these data to examine community structure. For the three cruises in 1991, 1993 and 2023, we identified which pairs of seabird species associate with one another more often than expected, based on the overall prevalence of those species within our survey area. As in Monier *et al.* [27], we tested for associations between every possible pair of species at a resolution of approximately 18.5 linear km (approx. 1 hour bins, after averaging the port and starboard observations). We chose a large bin size for our analyses because social cues can be useful in locating even faraway patches (that is, birds do not need a direct line of sight to the prey patch itself as long as they can see other birds flying in that direction [1,5,57,58].

Last, we calculated the Spearman rank correlation between species' abundances and the number of pairings with which each species engaged. We wanted to rule out whether species associations existed purely owing to the prevalence of the species within the areas surveyed.

## 3. Results

### (a) Seabird and marine mammal sightings (community change)

We identified 24 species of birds (belonging to seven families and four orders) and seven species of marine mammals (five families, two orders) sighted during at least one of the four survey years. Procellariiforms were consistently the most abundant taxon across all years (figure 2, shown in green); however, in 2023 diving-petrels (overwhelmingly common diving-petrels) were proportionally more dominant than in previous years. In 1985, the most numerous predators were prions (overwhelmingly Antarctic prions), pintado petrels and Antarctic fulmars (figure 2). In 1991, these changed to diving-petrels, pintado petrels and Antarctic fur seals (figure 2). In 1993, the dominant species were pintado petrels, blue petrels and diving-petrels (figure 2); in 2023, the most prevalent species were diving-petrels, Antarctic fur seals and giant petrels (table 1; figure 2). The only species included within the top four most prevalent species in all surveys was the pintado petrel. Pintado petrels are common throughout the Antarctic and subantarctic [59] and are well known to attend mixed-species foraging flocks [60,61]. The biggest changes over the 38-year period were the almost complete loss of Antarctic prions. Considering all our 2023 observations (which include some data outside of the east-end survey transects), we also observed dramatic increases in blue petrels and, especially, Kerguelen petrels, modest increases in king and gentoo penguins and snow and Antarctic petrels. There were substantial increases in all baleen whales. When counting all whales seen at all distances from the ship, 47 whales were sighted in 2023 (southern right, humpback and fin whales) compared with two southern right whales in 1993, no whales in 1991 and a single southern right whale in 1985.

**Figure 2.**
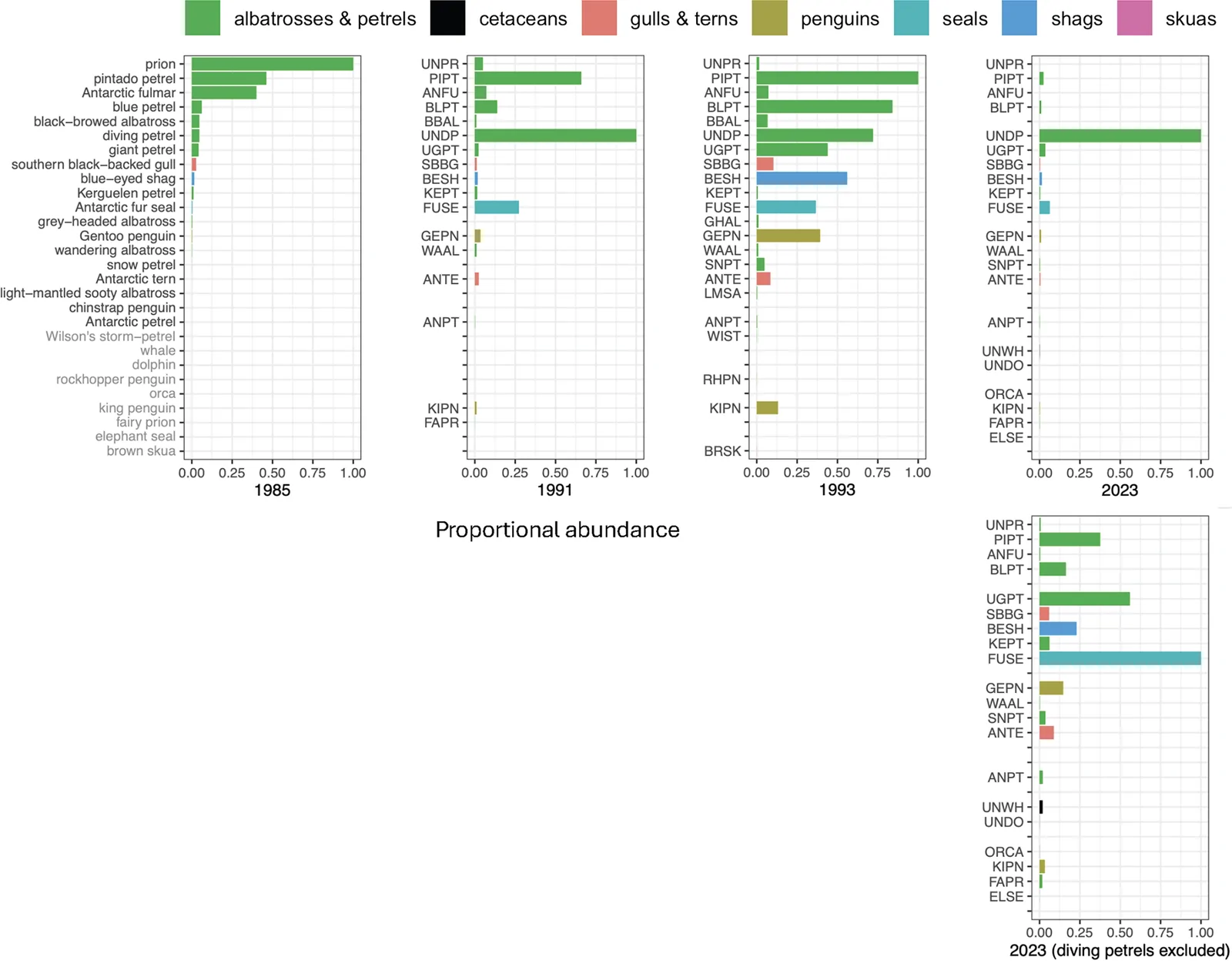
Community change over time. Mean daily individuals/km, standardized against the highest value for each year. Species are ordered based on 1985 abundances. Full common names are given for 1985 but abbreviated in subsequent years. Additionally, see table S1 in the supplementary material for full common and Latin names. Species not sighted in 1985 appear in a lighter text. In all other years, species not sighted are left blank on the axis. Diving-petrels were excluded from the bottom panel to better visualize the less abundant species. The 1985–1993 portion of this figure was originally published in Monier *et al*. [27], and is reproduced here with slight modifications under the terms of the Creative Commons Attribution License. See figure S1, in the supplementary material, for a version of this figure annotated to show abundances.

**Table 1.** Species sighted each year (a–c), their corresponding abundances and the number of significant RGA pairings they formed. Species are ordered by abundance; while we report the results of Spearman's rank showing positive correlations between species' abundance and the number of pairs formed, RGA significance was not limited to the most abundant species. Species not boldfaced did not form any pairings. To more easily compare across years, see table S1 in the supplementary material, which shows 1985–2023 abundances +/– SE.

a) 2023, Spearman's rho = 0.67 *S* = 505, *p* = 0.0008 (with diving-petrels excluded: Spearman's rho = 0.64, *S* = 481, *p* = 0.002)		
species	mean daily individuals/km	number of associations
**diving-petrels**	**20.74**	**4**
**Antarctic fur seal**	**1.31**	**1**
**giant petrels**	**0.73**	**4**
**pintado petrel**	**0.49**	**4**
blue-eyed shag	0.30	0
**blue petrel**	**0.21**	**7**
gentoo penguin	0.19	0
**Antarctic tern**	**0.12**	**5**
**Kerguelen petrel**	**0.08**	**1**
southern black-backed gull	0.08	0
**snow petrel**	**0.05**	**1**
king penguin	0.04	0
**Antarctic petrel**	**0.03**	**1**
whales	0.03	0
fairy prion	0.02	0
prions	0.01	0
Antarctic fulmar	0.01	0
wandering albatross	<0.01	0
dolphins	<0.01	0
orca	<0.01	0
southern elephant seal	<0.01	0

b) 1993, Spearman's rho = 0.80, *S* = 353, *p* = 0.000008		
species	mean daily individuals/km	number of associations
**pintado petrel**	**1.60**	**8**
**blue petrel**	**1.34**	**5**
**diving-petrels**	**1.15**	**9**
**blue-eyed shag**	**0.90**	**1**
**giant petrels**	**0.70**	**8**
**gentoo penguin**	**0.63**	**9**
**Antarctic fur seal**	**0.58**	**2**
king penguin	0.21	0
**southern black-backed gull**	**0.17**	**5**
**Antarctic tern**	**0.14**	**6**
**Antarctic fulmar**	**0.12**	**5**
**black-browed albatross**	**0.11**	**4**
snow petrel	0.08	0
prions	0.02	0
grey-headed albatross	0.02	0
wandering albatross	0.01	0
Kerguelen petrel	0.01	0
Antarctic petrel	<0.01	0
light-mantled sooty albatross	<0.01	0
rockhopper penguin	<0.01	0
brown skua	<0.01	0
Wilson's storm-petrel	<0.01	0

**c) 1991**, Spearman's rho = 0.67, *S* = 272, *p* = 0.003		
species	mean daily individuals/km	number of associations
**diving-petrels**	**17.89**	**10**
**pintado petrel**	**11.80**	**10**
**Antarctic fur seal**	**4.88**	**11**
**blue petrel**	**2.48**	**10**
**Antarctic fulmar**	**1.27**	**10**
**prions**	**0.89**	**8**
gentoo penguin	0.63	0
**Antarctic tern**	**0.44**	**11**
**giant petrels**	**0.40**	**11**
blue-eyed shag	0.32	0
**Kerguelen petrel**	**0.27**	**9**
**southern black-backed gull**	**0.21**	**8**
king penguin	0.19	0
**wandering albatross**	**0.19**	**3**
**black-browed albatross**	**0.16**	**7**
Antarctic petrel	0.03	0
fairy prion	0.01	0

### (b) Seabird and marine mammal associations

We found that approximately 57% of taxa formed associations in at least one of the four survey years. We found a substantial decline in the number of these significant interspecific pairings of seabird species around South Georgia over time ( figures 3 and 4). Network connectivity decreased from approximately 40% in 1991 to 13% in 1993 and to 7% in 2023. The number of associations each species formed also decreased; of the species that formed pairs, the median number of links decreased from 10 in 1991 (range 3–10, *n* = 12 pairing species) to 5 in 1993 (range 1–9, *n* = 11) and 4 in 2023 (range 1–7, *n* = 9).

**Figure 3.**
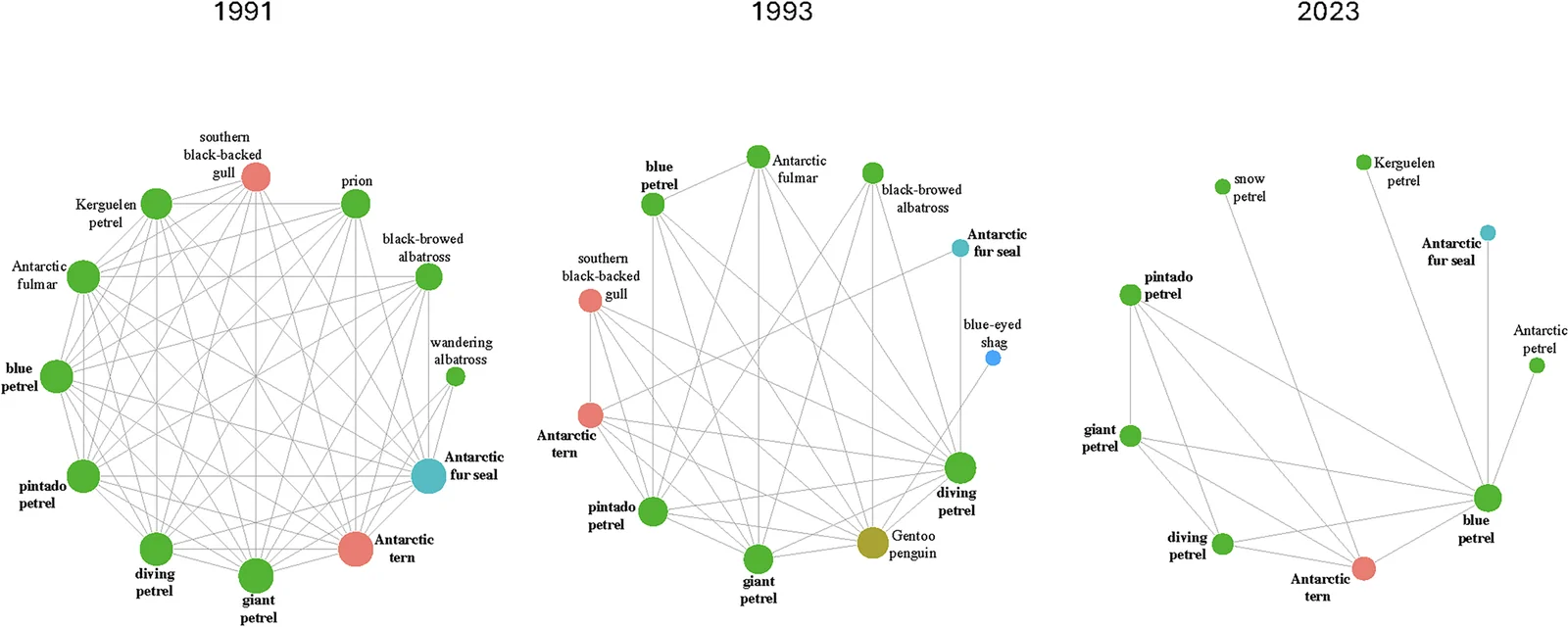
RGA results for 1991, 1993 and 2023. Lines link associated species. Node size is proportional to the number of pairs a species formed, with bigger circles indicating more associations. Species are arranged clockwise by connectivity, beginning around 5:00 (with Antarctic fur seal, diving-petrel and blue petrel forming the most associations in 1991, 1993 and 2023, respectively). Bolded species formed associations in all three years. Species are coloured by taxonomy using the same colour scheme as figure 2.

**Figure 4.**
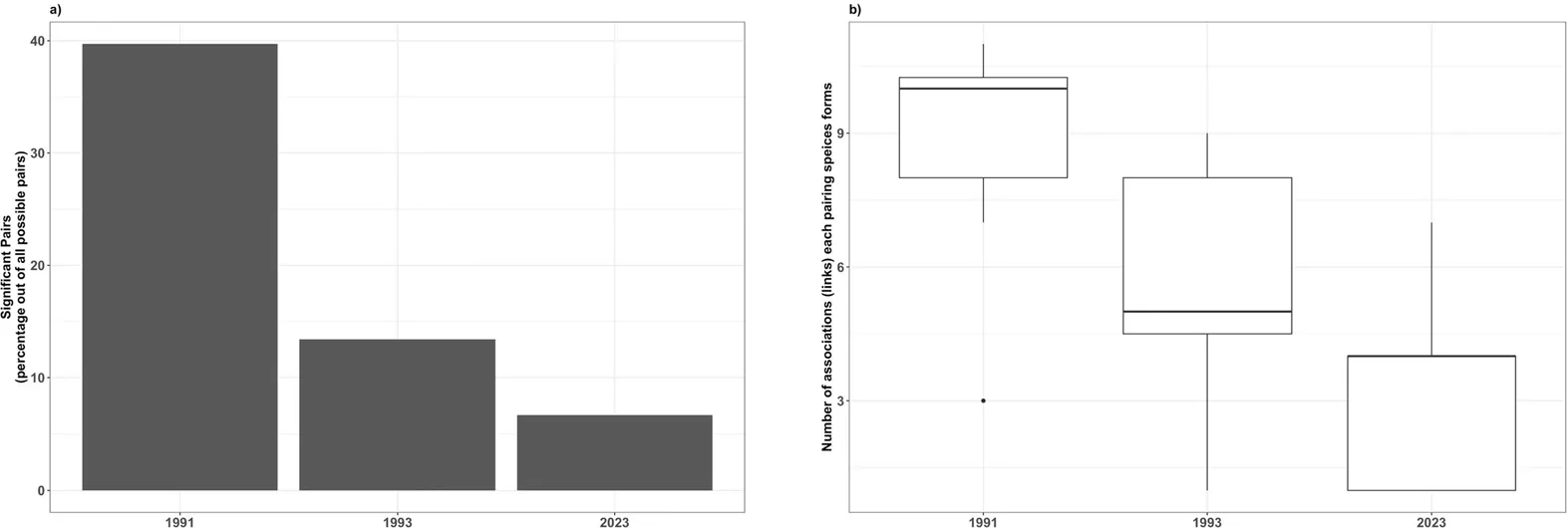
(a) Species pairings (percent of all possible pairs). (b) Number of associations formed by each pairing species.

We found a positive correlation between the abundance of each species and the number of significant pairings for every year (table 1, Spearman rank correlation of 0.64–0.80, with *p*-values on the order of 10^–3^ to 10^–6^). For 2023, this result holds even when the most numerous species group—diving-petrels—is removed (Spearman rank, *p* = 0.002). Although higher abundances did lead to more pairings (table 1), a more nuanced view is necessary as there are clear exceptions. Not all species with high abundance form significant associations (blue-eyed shag in 2023, king penguin in 1993 and other abundant species in table 1, all years). Further, species with very low abundances—black-browed albatross in 1991, Antarctic petrel in 2023—did form significant associations (table 1, parts a and c). In a given year, the most abundant species was never the one to form the most pairs.

## 4. Discussion

Our analysis supports our framework, indicating that the at least partially climate-related declines [27] of some species could lead to reduced opportunities for LE, and therefore for acquisition of food, by community members. Our results were in fact stronger than expected: the number of significant pairings among predator species dropped from 39.7% in 1991 to 6.7% in 2023. The implication of this finding is that, on top of the myriad threats that seabirds face [19,26], declines of individual species may be hastened because of reduced opportunity to locate patches of prey stemming from declines of other species whose foraging flocks they had previously joined.

### (a) Methodological limitations

Ship-based surveys are, of course, not without limitations. While our surveys were conducted during daylight hours, night-time foraging also occurs, and we were unable to document such events. Additionally, while ship surveys are better at detecting seabirds, particularly when it comes to smaller birds, aerial surveys may be better at detecting marine mammals [62]. All such surveys probably underestimate swimming species (such as marine mammals) that are only visible when they are near the surface. Despite such limitations, however, we nevertheless report an increase in marine mammals and detected seals forming associations in all years for which we had data.

RGA tests for statistically significant species pairings to reveal positive associations, but it is not a measure of LE usage *per se*; spatial co-occurrence can be the *result* of foraging interactions or it may just provide the *opportunity* for foraging interactions, and we cannot tease apart this difference in our data at this time. An additional limitation of RGA is that it does not test for associations between individuals of the same species. Intraspecific foraging associations are also an important component of seabird social foraging and LE, however [5,14,17,63–65], and therefore our study may actually under-report the decline in seabird foraging associations (if both associations between conspecifics and heterospecifics had been assessed).

Without the use of video cameras or tracking devices, it is difficult to follow individuals over extended periods at sea. Therefore, we tested for associations at the species level and cannot comment on the extent to which *individuals* form associations and use LE. We reason, however, that the majority of birds at sea are either searching for food or would opportunistically forage if they came upon a group of other foraging individuals.

### (b) Community change and reduced opportunities for local enhancement

We continued our time series of seabird surveys begun in winter 1985 at South Georgia, to ask whether the documented declining abundance of some predators has led to reduced opportunities for LE among pelagic foraging vertebrate predators. As the majority of the species recorded across all our surveys (approx. 57%) readily join mixed-species foraging aggregations [35], it seems likely that our projection of lower foraging success is reasonable. In this paper, we show that the number of positive species associations has decreased over time, with networks showing both fewer links and fewer nodes (figures 3 and 4). Thus, a decline in abundance of individual species (represented by community change, figure 2) would probably lead to reduced opportunities for LE.

The framework presented in [27] predicts that species found in winter at South Georgia would decline because of lost foraging opportunity. But not all species there have declined, even though increasing sea temperatures and perhaps overharvesting have led to declines in the abundance of Antarctic krill [25,40,66–68]. Ecological relationships are complicated at South Georgia owing to the combined and often conflicting impacts of climate change (including breakup of sea ice), the krill commercial fishery and the continued increases in whales and seals owing to cessation of commercial hunting. Baleen whales have certainly increased in frequency over our period of study [42,69–73], and while we did not observe many feeding birds associated with whales in 2023, we saw many more whales than in previous years.

Further complicating this system is the continued breaking up of glaciers in the Antarctic [66,74]. The 100 km-long iceberg ‘A-76’ [74] was aground off the eastern end of South Georgia during our cruise in July 2023; it gradually broke up and continued to drift to the west and eventually north during our sampling period. Most of the snow and Antarctic petrels that we saw in 2023 were directly associated with this iceberg and pieces that had broken off from it. These petrels are cold-water, high Antarctic species that we would have expected to be *less* common than on our previous cruises, and their rather unexpected presence, in light of warming waters, was clearly owing to the presence of this Weddell Sea iceberg. Large icebergs are making the waters around South Georgia colder, less saline [75–77] and less conducive to krill recruitment [78]. In this way, the presence of icebergs in 2023 may have been masking otherwise ongoing environmental changes to the area; without the influx of cold water from these icebergs, sea surface temperatures would probably have been warmer, for example.

We present community and abundance data as a potential mechanism for the changes in associations displayed in figure 3. There are several explanations that might fit the changes in associations, however. One explanation is that the most abundant species, purely through larger abundances, are more likely to form significant pairings. We did check for an association between species abundances and the number of significant pairings for each species. Although we found a positive Spearman rank correlation, the statistical explanation that this suggests is not the only driver in this system (see the high abundance species that failed to pair and the low abundance species with many pairings listed in table 1).

It might also be that there is more impetus to form significant associations with other species when prey availability is low. Social feeding is a tool that should increase one's chance of finding food, and therefore if prey availability is high, it is less important to use other species as cues to find prey. A modelling study by Bastos *et al.* [79] compared the use of olfactory cues with LE and found that when prey conditions were good, the two search strategies were equally effective. Additionally, an at-sea experiment using wild individuals found that common murres (*Uria aalge*) use LE less often when prey could otherwise be reliably located nearby [80]. Thus, the decline in connections (from 1991 to 2023) could possibly be an indication that prey availability is changing for the better rather than (or in addition to) reflecting changing abundances in the community. However, recent work shows that this is not the case. Kawaguchi *et al.* [25] reviewed the literature and found that Antarctic krill populations are declining. Further, Abram *et al.* [81] show that a widespread regime shift is underway in Antarctica, driven by climate change and resulting in lowered resources to support krill recruitment.

Who associates with whom might be owing to diet (e.g. squid-eaters associating with other squid-eaters). Alternatively, large feeding flocks with many birds and mammals are very conspicuous, and foragers that see these aggregations may be drawn to the patch to investigate before they ‘know’ which prey items are available. Regardless, this does not change our main finding, which is that the tendency to form mixed-species associations has changed over time. If LE were not being used, each individual would have to find and arrive at the patch independently—and since flying birds and foraging aggregations are visible from a much greater distance than the patch itself [5], we believe this is unlikely, especially because patch location (assessed by the direction in which another forager is coming/going), feeding attempts (dives) and feeding success (fish in mouth) are public information. In other systems, prey partitioning is strong, and which predators are found together in a feeding flock has been shown to be characterized by the prey that those species consume [82–84]. Prey (i.e. diet) diversity in the Antarctic is low, however, especially compared with other oceans [82,85]. Krill is either explicitly targeted or opportunistically consumed by the overwhelming majority of the species we observed; fish and squid are also important prey items, but these other prey items can consume krill themselves [82,86–88], resulting in mixed-species prey patches. A mixed-species prey patch would then attract a mixture of birds that could be targeting krill or krill-eating fish and squid.

Blue petrels, and especially Kerguelen petrels, are generally not found in mixed-species foraging aggregations [59], although we found that blue petrels did form pairs in all years and, in fact, they formed more associations than any other species in 2023. Blue petrels commonly associate with prions (they have similar plumage, which means that they can be difficult for humans to distinguish from one another, and this morphological and ecological similarity might make them useful foraging cues to one another), but there were hardly any prions present to associate with the blue petrels in 2023. Snow petrels and Antarctic petrels sometimes join mixed-species foraging aggregations, but they are both also closely linked to icebergs [89]. The edge of the pack ice and the presence of icebergs in 2023 probably influenced their separation from other species. In 2023, it was a surprise that Antarctic fur seals did not form pairs with any other species of predator than the blue petrel because they often act as an initiator for mixed-species aggregations [35]. This result may be driven by the fact that most of the fur seals we saw were in just a small handful of large aggregations.

### (c) Future directions

Further research to determine whether dietary shifts are driving the composition of feeding aggregations would be very useful. Second, there is some ambiguity about the consistency of the use of LE by certain species. The species joining mixed-species flocks seems to vary between years, at least in the California current [90], the Hauraki Gulf near the North Island of New Zealand [84] and between seasons in the Humboldt current [83]. Individual birds may join or not join mixed-species flocks depending on what prey are available and how abundant these prey are in any given year. Thus, prey availability should be examined as a possible driver of species' tendencies to form significant pairings. Last, although much remains unknown about tendency to pair, and with whom species pair, the impact of declining LE opportunities should be considered when making management decisions for Antarctic seabirds. However, the general lack of published data regarding this winter community makes it difficult to assess these populations. Additional winter surveys (additional years of data spanning more of the winter season) are necessary for a more complete picture of the abundance and distribution of these species outside of just the breeding season.

## Supplementary Material



## Data Availability

Data files and R code are provided in the supplementary material [91].

## Theme

One contribution of 14 to a theme Issue ‘Multi-predator feeding aggregations in pelagic waters’.
