# Supplementary material for: Changes in species composition of South Georgia seabirds and mammals 1985–2023: decrease in opportunities for local enhancement?
Source: Philos Trans R Soc Lond B Biol Sci. 2026 Oct 15;381(1961):20250363. doi: 10.1098/rstb.2025.0363 (PMC13670073; doi:10.1098/rstb.2025.0363)
Supplement: Supplementary file 1 [file rstb.2025.0363.zip › rstb.2025.0363/rstb20250363_si_002.pdf]

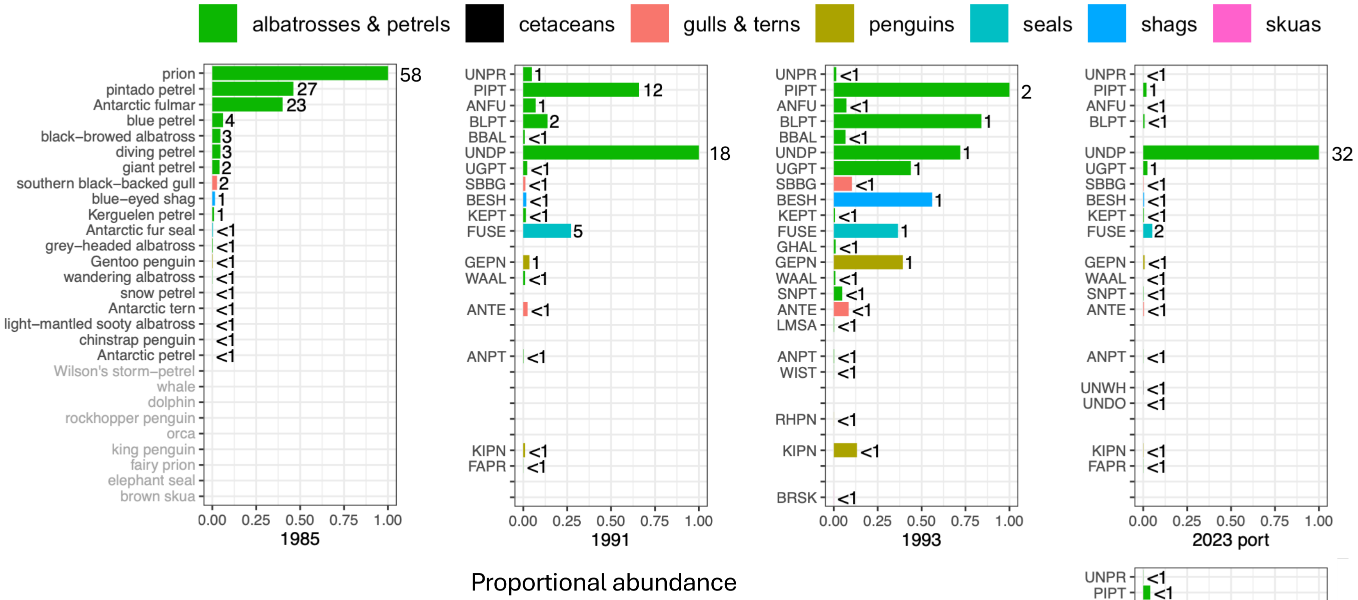

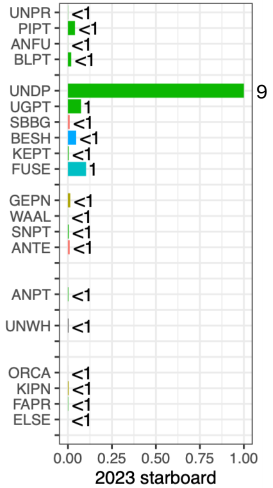

Figure S1. This figure is identical to Figure 2 in the main text, but annotated to show abundance (mean daily individuals/km, rounded to the nearest whole number) to the right of each bar. Additionally, port and starboard values are shown separately for comparison. Bar lengths are standardized against the highest value for each year. Species are ordered based on 1985 abundances. Full common names are given for 1985 but abbreviated in subsequent years. Species not sighted in 1985 appear in a lighter text. In all other years, species not sighted are left blank on the axis.

Monier S, Manne LL, Veit RR. Changes in recurrent groups of South Georgia seabirds and mammals 1985–2023: decrease in opportunities for Local Enhancement? Philos Trans R Soc B.
